# Feasibility of neck electrical impedance tomography to monitor upper airway dynamics during sleep

**DOI:** 10.3389/frsle.2023.1238508

**Published:** 2023-09-21

**Authors:** Vivien S. Piccin, Erick D. L. B. de Camargo, Rafaela G. S. Andrade, Vinícius Torsani, Fabíola Schorr, Priscilla S. Sardinha, Fernanda Madeiro, Pedro R. Genta, Marcelo G. Gregório, Carlos R. R. de Carvalho, Marcelo B. P. Amato, Geraldo Lorenzi-Filho

**Affiliations:** ^1^Sleep Laboratory of the Heart Institute (InCor), University of Sao Paulo Medical School, São Paulo, Brazil; ^2^Biomedical Engineering, Center for Engineering, Modeling and Applied Social Sciences, Federal University of ABC, São Bernardo do Campo, Brazil; ^3^Respiratory ICU, Hospital das Clinicas, University of Sao Paulo Medical School, São Paulo, Brazil

**Keywords:** diagnostic imaging, electric impedance, obstructive sleep apnea, structure collapse, electrical impedance tomography, nasofibroscopy

## Abstract

**Background:**

There is a lack of non-invasive methods for monitoring the upper airway patency during sleep. Electrical impedance tomography (EIT) is a non-invasive, radiation-free tool that has been validated to monitor lung ventilation. We hypothesized that electrical impedance tomography (EIT) can be used for monitoring upper airway patency during sleep.

**Methods:**

Sleep was induced in 21 subjects (14 males, age 43 ± 13 years, body mass index 32.0 ± 5.3 kg/m^2^) with suspected obstructive sleep apnea (apnea-hypopnea index: 44 ± 37 events/h, range: 1–122 events/h) using low doses of midazolam. Patients wore a nasal mask attached to a modified CPAP device, allowing variable and controlled degrees of upper airway obstruction. Confirmation of upper airway patency was obtained with direct visualization of the upper airway using nasofibroscopy (*n* = 6). The changes in total neck impedance and in impedance in four cranio-caudal regions of interest (ROIs) were analyzed.

**Results:**

Total neck impedance varied in concert with breathing cycles and peaked during expiration in all patients. Group data showed a high cross-correlation between flow and impedance curves (*r* = −0.817, *p* < 0.001). Inspiratory peak flow correlated with simultaneous neck impedance (*r* = 0.866, *p* < 0.001). There was a high correlation between total neck impedance and velopharynx area (*r* = 0.884, *p* < 0.001), and total neck impedance and oropharynx area (*r* = 0.891, *p* < 0.001).

**Conclusions:**

Neck EIT is sensitive and captures pharyngeal obstruction under various conditions. Neck EIT is a promising method for real-time monitoring of the pharynx during sleep.

## Introduction

Obstructive sleep apnea (OSA) is characterized by repetitive pharyngeal collapse during sleep, resulting in recurrent arousals and arterial oxygen desaturation (Young et al., 1993; Tufik et al., 2010). Upper airway collapse results from complex interaction of multiple factors, including respiratory control instability, insufficient pharyngeal muscle dilatory activity and upper airway anatomy (Haponik et al., 1983; Suratt et al., 1983; Schwab et al., 1995; Mezzanotte et al., 1996; Heinzer et al., 2005). In OSA patients, recurrent obstruction occurs in the upper airway between the soft palate, the palatal tonsils, the base of the tongue and the lateral pharyngeal walls (Kim et al., 2019). On the other hand, the dynamic mechanisms leading to upper airway obstruction during sleep are poorly understood. Imaging techniques, such as computed tomography (CT) and magnetic resonance imaging (MRI), have provided detailed insights about the upper airway of patients while awake, but limited information during sleep (Abramson et al., 2010; Tang et al., 2012; Brown et al., 2013; Wang et al., 2014; Zhang et al., 2014). Direct visualization of the upper airway using drug-induced sleep endoscopy is an invasive method that has also provided only partial information regarding the dynamics of the upper airway during obstruction (Badr et al., 1995; Hewitt et al., 2009; Rodriguez-Bruno et al., 2009). Forced oscillation technique (FOT) is a non-invasive method to monitor upper airway patency, but the application requires a nasal mask and does not allow insights on anatomical behavior of the upper airway (Campana et al., 2012). Therefore, the monitoring of the dynamic upper airway collapse during sleep may provide a window of opportunity to better understanding the pathophysiology of OSA.

Electrical impedance tomography (EIT) is based on the concept that the injection of small amounts of electrical current (5–10 mA; 125 kHz) in a rotating sequence through pairs of surface electrodes provide electrical potentials that vary according to the shape and distribution of the anatomical area under study (Kim et al., 2019). EIT capacity to measure lung aeration (Victorino et al., 2004; Costa et al., 2009a) was validated previously since air has much higher impeditivity than tissues (Brown et al., 1985; Frerichs et al., 1999). Typically, 32 electrodes are positioned linearly around the thorax. This configuration is based on the Sheffield protocol (Brown and Seagar, 1987) and provides information regarding a total cross-sectional width of ~7–10 cm (Costa et al., 2009b).

Kim et al. (2019) conducted a study with seven healthy participants and 10 patients with OSA under non sedated sleep, to determine whether EIT could identify upper airway narrowing or collapse. In that study, transient varsupsetneqqairway closure was induced by the swallowing maneuver, and EIT images were confirmed by simultaneous magnetic resonance imaging (MRI) scans. Obstructive hypopnea and apnea were detected successfully by EIT in 10 patients with OSA, and no significant changes in EIT data were observed in seven healthy participants during concurrent EIT and PSG tests. Authors concluded that EIT could be a useful real-time monitoring device for detecting upper airway narrowing or collapse during natural sleep, in OSA patients.

Another study applying EIT imaging technique to evaluate upper airway was conducted by Ayoub et al. (2019), with seven healthy subjects (six male and one female) with no history of witnessed apnea and ten male OSA patients. The subject was connected to the PSG and the 16-channel EIT device at the supine position. For EIT imaging, electrical currents of 1 mArms at 11.25 kHz were sequentially injected between chosen pairs of neighboring electrodes and induced voltages were measured between other neighboring electrode pairs. After removing the artifact components, they demonstrated the feasibility of the upper airway EIT imaging technique to characterize obstructive hypopnea and apnea events during natural sleep. In that study, during normal breathing, EIT images clearly showed that the upper airway was totally open and filled with the air. The air was replaced by conductive upper airway soft tissues during obstructive hypopnea and apnea events, which were successfully detected in the reconstructed EIT images (Ayoub et al., 2019).

In the same way, Ayoub et al. (2020) analyzed time series of reconstructed EIT images, providing quantitative information about how much the upper airway was closed during collapse and reopening. Ten OSA patients' data were studied, and the results showed that the EIT can compare the upper airway dynamics between obstructive apnea and hypopnea.

Our work, the first one that uses EIT technique in induced sleep, was designed to study neck EIT as a feasible method of monitoring upper airway patency during sleep.

This study applies EIT on the upper airway under the hypothesis that electrical impedance tomography can be a continuous upper airway imaging technique during sleep. To this end, we studied patients with a wide range of OSA severity. To obtain variable degrees of upper airway obstruction we used the method of critical closure pressure (Pcrit) determination, applying variable levels of nasal pressure during induced sleep. In a sub sample we directly visualized the upper airway anatomy using endoscopy.

## Materials and methods

Subjects aged between 18 and 70 years suspected of having OSA referred to the Sleep Laboratory at the Heart Institute—Hospital das Clínicas were invited to participate. Subjects with previous upper airway surgery and significant heart or pulmonary disease were excluded. All participants underwent standard diagnostic overnight polysomnography (PSG) (Embla Systems Inc., USA) (Gamaldo et al., 2014). Subjects provided written informed consent, and the protocol was previously approved by the Hospital das Clínicas Ethics Committee (Protocol Number: 0748/11).

### Neck electrical impedance tomography

EIT is a method of estimating impedance distribution (or variations of impedance distribution) inside a domain. This domain is discretized using the Finite Element Method (FEM). The vector of impedance of each finite element represents the variations of the impedance distribution in time. The problem of estimating the vector of impedance based on the injected current, measuring electrical potentials on the boundary of the domain and knowing the structure of the model may be classified as an inverse problem. The algorithm is a sensitivity matrix. Two different sets of measured voltages are required: the first set (V0) is the reference set of measurements, and the second set (V1) is related to the modified impedance distribution. An estimate of the normalized resistivity distribution may be computed using the Equation (1), where “B” is the inverse of the sensitivity matrix, and ρ is the impeditivity (Δρ = (ρ*1-* ρ*0)/*ρ*0)* (Aya et al., 2007).


(1) $$\begin{array}{l} \Delta \rho =\text{B}\Delta \text{V} \end{array}$$

Because the image formation problem of EIT is a difficult inverse problem due to its non-linearity, regularization methods are required. For this project, a Spatial Gaussian Filter was used for regulation, resulting in smooth images (Aya et al., 2007).

**Figure 1:**
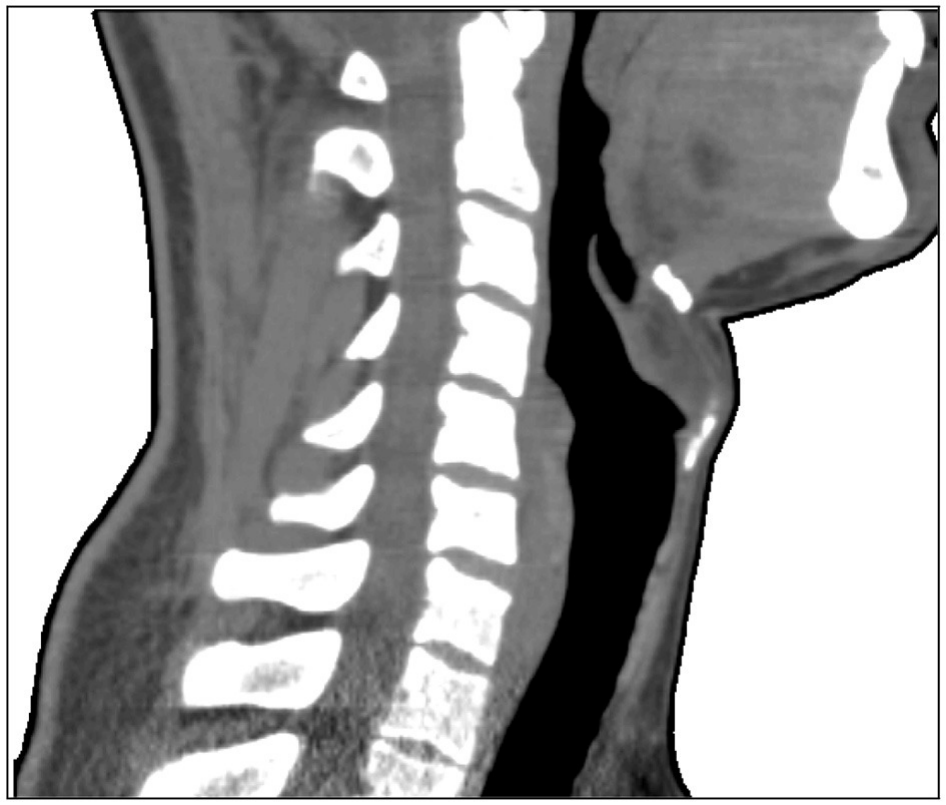
Sample CT-scan image of a volunteer, used to create a three-dimensional finite element mesh.

**Figure 2:**
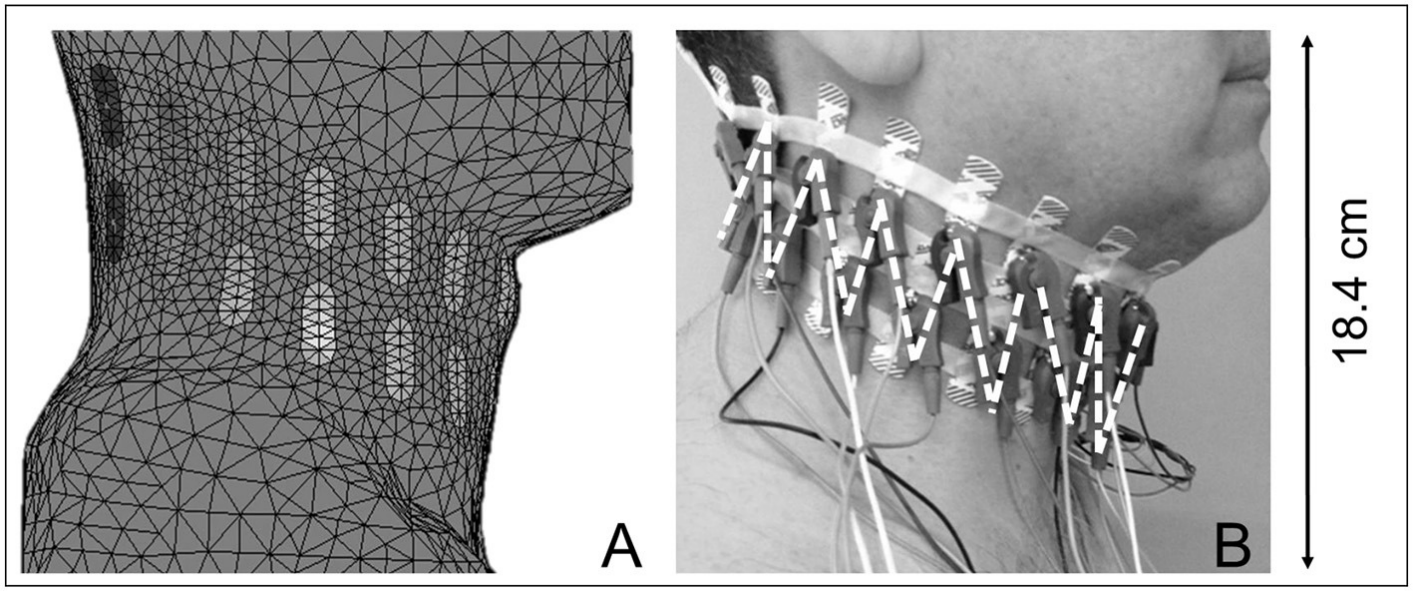
**(A)** Illustrates a finite element model of the neck. **(B)** Depicts a subject wearing 32 electrodes spanning his neck circumference in two equidistant bands, with 16 electrodes making up each band; the first strap is located near the level of the maxilla, and the second is immediately beneath the first. Small connectors were positioned in a zigzag configuration, as illustrated by the dashed lines. Neck EIT covers a vertical length of 18.4 cm.

A three dimensional (3D) finite element mesh was created based on CT-scan images of a volunteer (Figure 1). The resulting mesh contained 62,500 tetrahedral elements and 11,700 nodes, and its total dimensions were 184 × 221 × 153 mm (height × length × width) (Figure 2A). Thirty-two electrodes were placed around the subject's neck circumference in a zigzag configuration, allowing the determination of impedance variation across a vertical length of 18.4 cm (Figure 2B). The electrodes were connected to an impedance tomography device (Enlight^®^, Timpel, Brazil), which generates 50 images per second.

For each set of measured voltages, impeditivity changes (Δρ) were noted using the Equation (1) for all tetrahedral elements, and a cross-sectional image of the sagittal plane passing through the center of the mesh was created.

Small amounts of electrical current (5–10 mA; 125 kHz) were injected in a rotating sequence through pairs of electrodes, with one non-injecting electrode interposed between the injecting electrodes. These currents traveled through the neck following pathways that varied according to neck shape and the distribution of impeditivities, generating an electrical potential gradient at the surface, which was then transformed into a 3D image of the electrical impedance distribution within the neck. Surface electrical measurements were used to infer living tissue impeditivity. A low-pass filter (0.333 Hz) minimized perfusion interference. Image reconstruction was based on relative changes in impedance relative to a reference (first 300 frames of neck EIT), assuming that the shape of the neck did not change. Only regions of the upper airway in which the impedance changed over time were represented among the EIT images (Costa et al., 2009b). In this study the changes in total neck impedance and in impedance in four cranio-caudal regions of interest (ROIs) were analyzed. The total neck impedance value, as well as the ROI values, were obtained through the sum of the pixels values of the image (or the respective ROI).

### Data acquisition during sleep

The subjects remained in the sleep laboratory in the morning immediately after diagnostic polysomnography (PSG). Sleep was induced using low doses of midazolam (Genta et al., 2011) with the subjects used a nasal mask connected to a modified CPAP device (Philips Respironics, Murrysville, PA) interposed by 2 pneumotachographs connected in series. The CPAP was initially titrated to overcome obstructive events and flow limitations during sleep (holding pressure). Pcrit was determined as previously described (Gleadhill et al., 1991). Briefly, once stable stage 2 was achieved at the holding pressure for at least 2 min, the CPAP was abruptly reduced by 1 cmH_2_O during expiration and was held at this level for five breaths. CPAP was then returned to the holding pressure for 1 min before being dropped an additional 1 cmH_2_O for another five breaths. This process entailed progressive CPAP decreases until obstructive apnea occurred. Flow and pressure curves were analyzed using custom-designed software (Matlab, The MathWorks, Natick, MA). Neck EIT data were stored in a computer that recorded neck impedance and pressure and flow measurements derived from the second nasal mask pneumotacograph. Neck EIT images and inspiratory peak flow (V'Imax) were analyzed using custom-designed software (LabVIEW, National Instruments Corporation). Pcrit determination allowed for neck EIT assessments under controlled levels of flow limitation.

### Direct measurement of the upper airway

Sleep endoscopy was performed following Pcrit determination in a subgroup of patients (*n* = 6). An ultra-slim bronchofibervideoscope (2.8 mm diameter, Olympus^®^ BF type XP160F) was inserted through a sealed port in the nasal mask. The distance between the tip of the scope and the area of interest was measured using a wire (marked in centimeters) that passed through the aspiration channel of the scope (Schorr et al., 2012). The scope's tip was placed one centimeter above the velopharynx (VP: retropalatal airway) and one centimeter above the oropharynx (OP: retroglossal airway). Images were digitally recorded during five breaths under the following 3 different nasal pressures: with no flow limitation, with flow limitation and during obstructive apnea. At each level of nasal pressure, the images of the smallest VP and OP areas of one representative respiratory cycle were captured using specific software (Vegas Movie Studio HD Platinum 11.0, Sony Creative Software Inc.). The area was calculated by delimiting the lumen image (ImagePro Plus 4.5.0.19, Media Cybernetics Inc.) and comparing it with the same distance-magnification using millimeter paper (Isono et al., 2002). VP and OP areas were plotted against values of impedance valleys collected at the holding pressure, under flow limitation and during obstructive apnea.

### Statistical analysis

Data were expressed as means ± SD (or medians when appropriate). Mann–Whitney *U*-test was used to detect the differences in the males' and females' baseline characteristics, sleep study, CPAP holding pressure and Pcrit. The relationship between flow and total impedance changes during stable breathing was tested using cross-correlation. We used the Fischer *r*-to-*z* transformation to transform these correlation coefficient values into weighted additive quantities. Spearman's rank correlation coefficient was applied to correlate V'Imax and impedance at each step of the pressure reduction during Pcrit determination. Also the Spearman's rank correlation coefficient was applied to correlate the mean impedance value in the impedance valley with the smallest VP and OP cross-sectional areas at nasal pressures with no flow restriction, with flow restriction and during apnea. Because subjects' levels of nasal CPAP were different and because impedance is a relative value, the *z*-score transformation [*z*-score = (*x* – μ)/σ, where *x* = sample value, μ = sample mean, and σ = standard deviation] was used to normalize and correlate the pooled data of V'Imax, neck impedance and pharyngeal area. Thereafter, we used a generalized estimation equation (GEE) model to determine in a subgroup of patients (*n* = 6) the numerical relationship between total neck impedance and the minimum VP and OP areas. The Kruskal-Wallis test was employed to identify differences in impedance variation between ROIs (delta between the mean impedance values at three consecutive peak inspiratory flow measurements) during stable breathing and severe flow restriction. The Wilcoxon test was used to define which ROI exhibited the higher delta value. Statistical significance was set at *p* < 0.05. SPSS 15.0 for Windows^®^ was used (2006 SPSS Inc., Chicago, Illinois).

## Results

Twenty-one individuals completed the study (Table 1).

**Table 1:** Baseline characteristics, sleep study, CPAP holding pressure and Pcrit of the population studied.

**No. total of subjects**	**21**		
	**Male**	**Female**	**Total range**
No. by gender, %	14 (67%)	7 (33%)	
Age, years	40 ± 13	50 ± 9	23–69
BMI, kg/m^2^	32 ± 5	32 ± 5	24–43
Neck circumference, cm	42 ± 3	38 ± 2	36–50
AHI, events per hour	44 ± 37	44 ± 35	1–122
Min SaO_2_, %	81 ± 10	82 ± 6	52–91
ESS	11 ± 5	14 ± 6	2–22
CPAP holding pressure, cmH_2_O	13 ± 4	14 ± 3	5–20
Pcrit, cmH_2_O	2 ± 5	4 ± 3	−4 to 10

Values are expressed as the means ± SD. CPAP, continuous positive airway pressure; Pcrit, critical closing pressure; BMI, body mass index; ESS, Epworth Scale; AHI, apnea-hypopnea index. The statistical analysis found no significant differences between genders.

**Figure 3:**
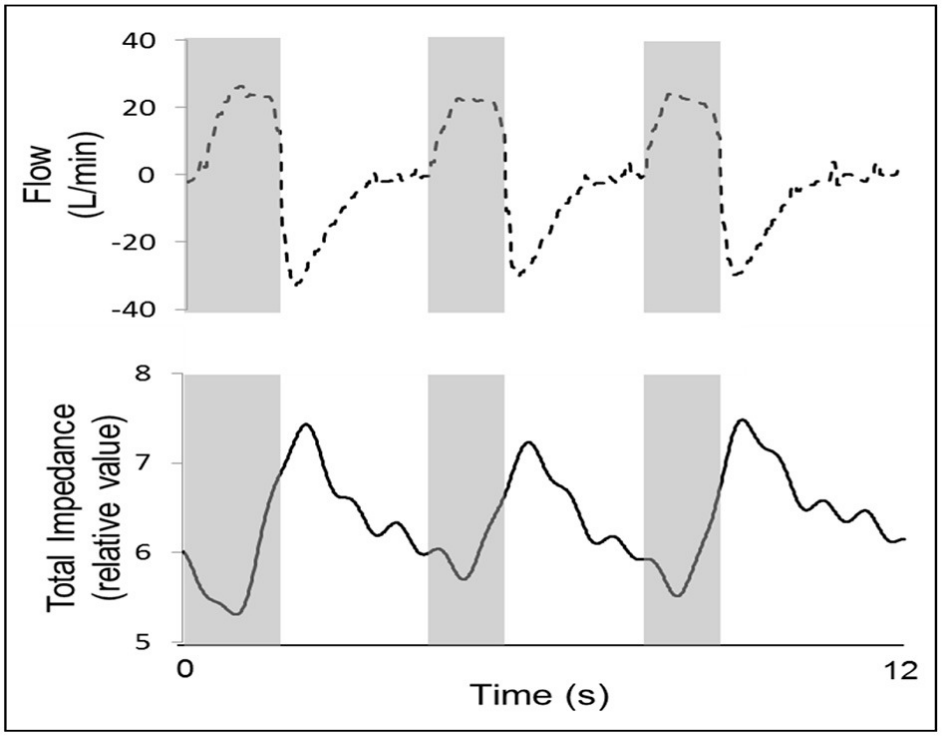
An example of one representative subject, showing that total impedance (solid line) varies during stable breathing (presented by a flow curve, dashed line). During measurements of the impedance during stable breathing, valleys occurred during inspiration (gray columns), and impedance peaks occurred during expiration. Because air is a poor conductor of electricity, the lower value of the upper airway cross-sectional area occurred during inspiration, and the higher value occurred during expiration. In this example, *r* = 0.914, *p* < 0.001. Flow data was acquired by the EIT machine a pneumotachograph connected in series with the airflow circuit, which saves the flow data synchronously with the impedance data.

Sleep was induced with a mean total midazolam dose of 2.5 ± 1.2 mg. In all subjects, the total impedance varied within the respiratory cycle during stable breathing, as shown in a representative example (Figure 3).

**Figure 4:**
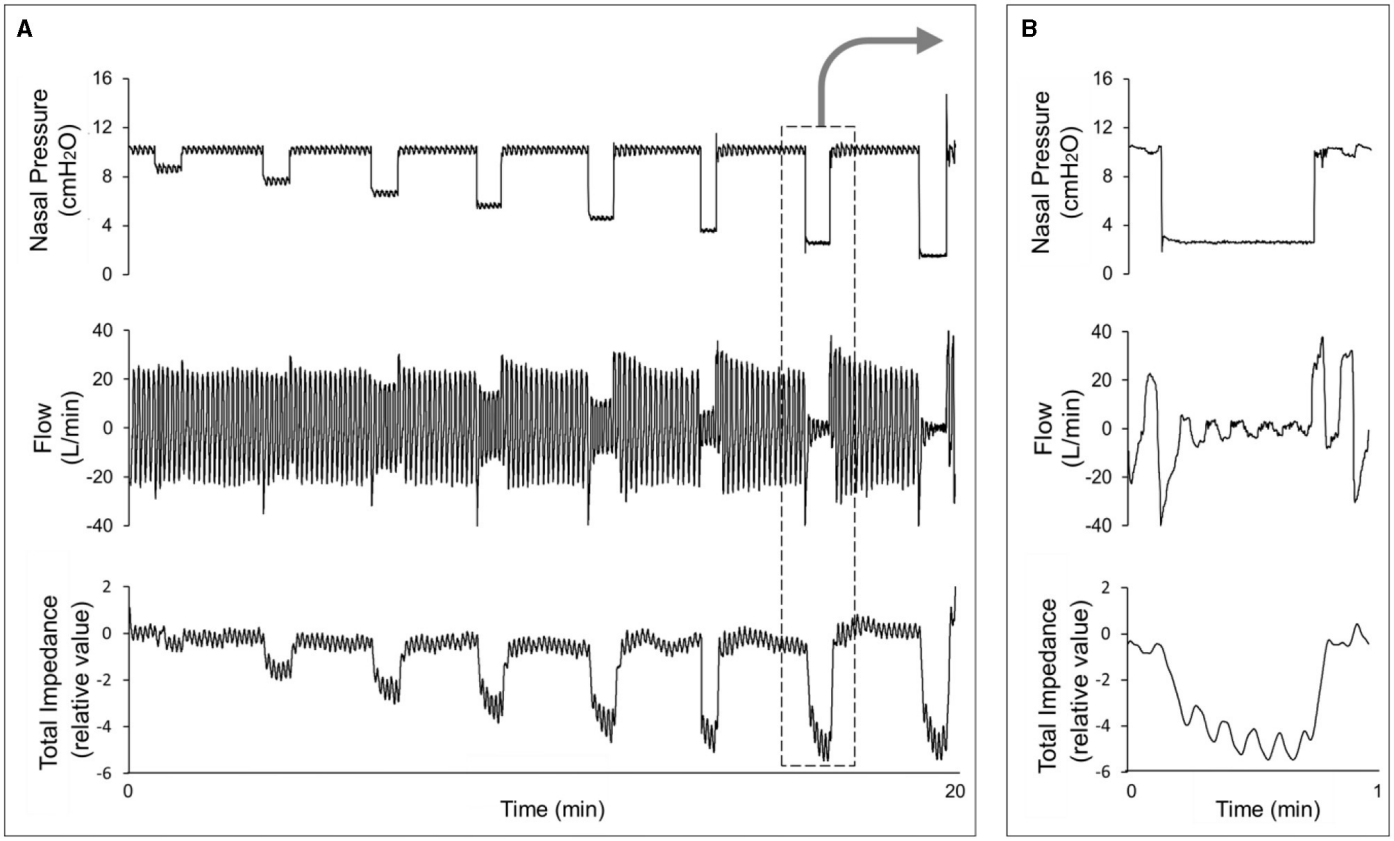
**(A)** Shows an example from a representative subject in whom we observed a flow decrease, followed by impedance variation, during CPAP reduction. **(B)** Provides a magnification of one step of the CPAP reduction (arrow), showing that the impedance also varies during the breathing cycle.

In all cases, the impedance valleys were close to the inspiratory peak flow, and the impedance peaks were close to the expiratory peak flow. During stable breathing, the group demonstrated an average cross-correlation between the flow and impedance curves of *r* = −0.817, obtained during stable breathing (*p* < 0.001). The mean time-lag was 0.48 ± 0.35 s (range: 0–1.52 s). During nasal pressure reductions, we observed that the total neck impedance decreased in proportion to the flow reduction on all occasions and in all patients, as observed in a representative subject (Figure 4).

Table 2 demonstrates that the delta between the average impedance values of three consecutives peak inspiratory flow measurements taken during stable breathing and during severe flow restriction was significantly higher in the 2nd ROI than in the 1st, 3^rd^, and 4th ROIs.

**Table 2:** Delta between the impedance determined during stable breathing and impedance during severe flow restriction.

	**Delta**	**Range**
1st ROI	−2.049 ± 1.240	−4.081/−0.497
2nd ROI	−3.286 ± 1.895^*^	−6.482/−0.907
3rd ROI	−1.980 ± 1.382	−4.803/−0.097
4th ROI	−1.684 ± 1.527	−3.776/+1.539

Group data for the delta between the mean impedance value at three consecutive inspiratory peak flows, during stable breathing and during severe flow restriction. The impedance delta was significantly higher in the 2nd region of interest (ROI) compared with the remaining ROIs (*p* < 0.001). The results are expressed as means ± SD. ROI: region of interest (*n* = 21). ^*^p < 0.001.

**Figure 5:**
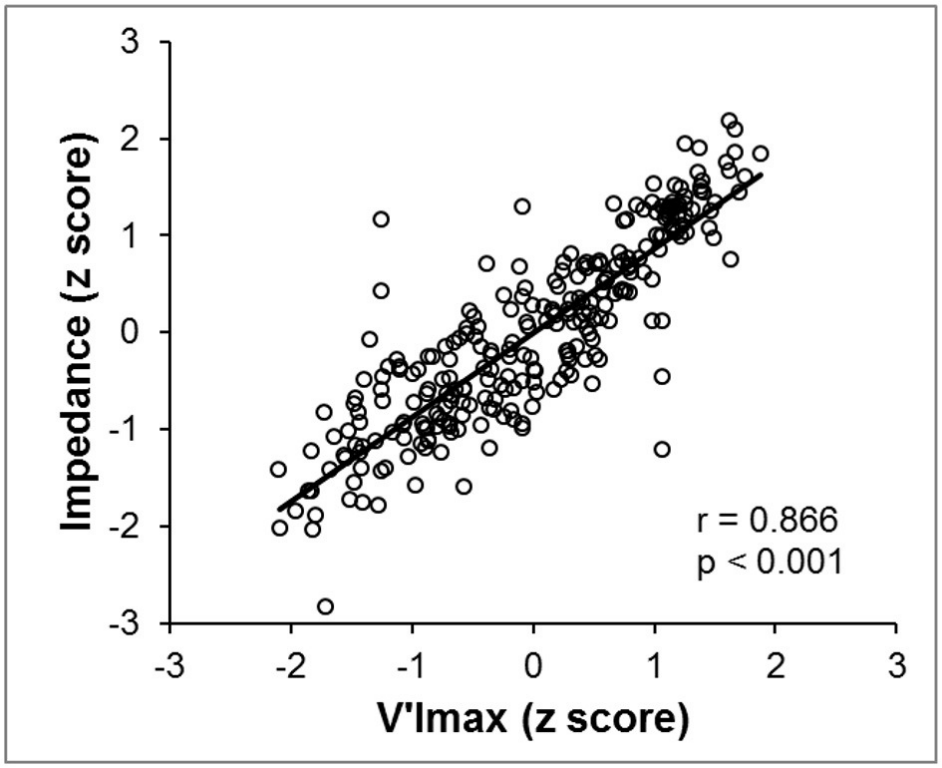
In all subjects, we observed a high correlation coefficient between the total neck impedance variation *z*-scores (*Y*-axis) and the V'Imax *z*-scores (*X*-axis). Data were acquired while the pressure was reduced until apnea occurred.

V'Imax and total neck impedance (expressed as *z*-score) were strongly correlated in the entire group (*r* = 0.866, *p* < 0.001) (Figure 5).

**Figure 6:**
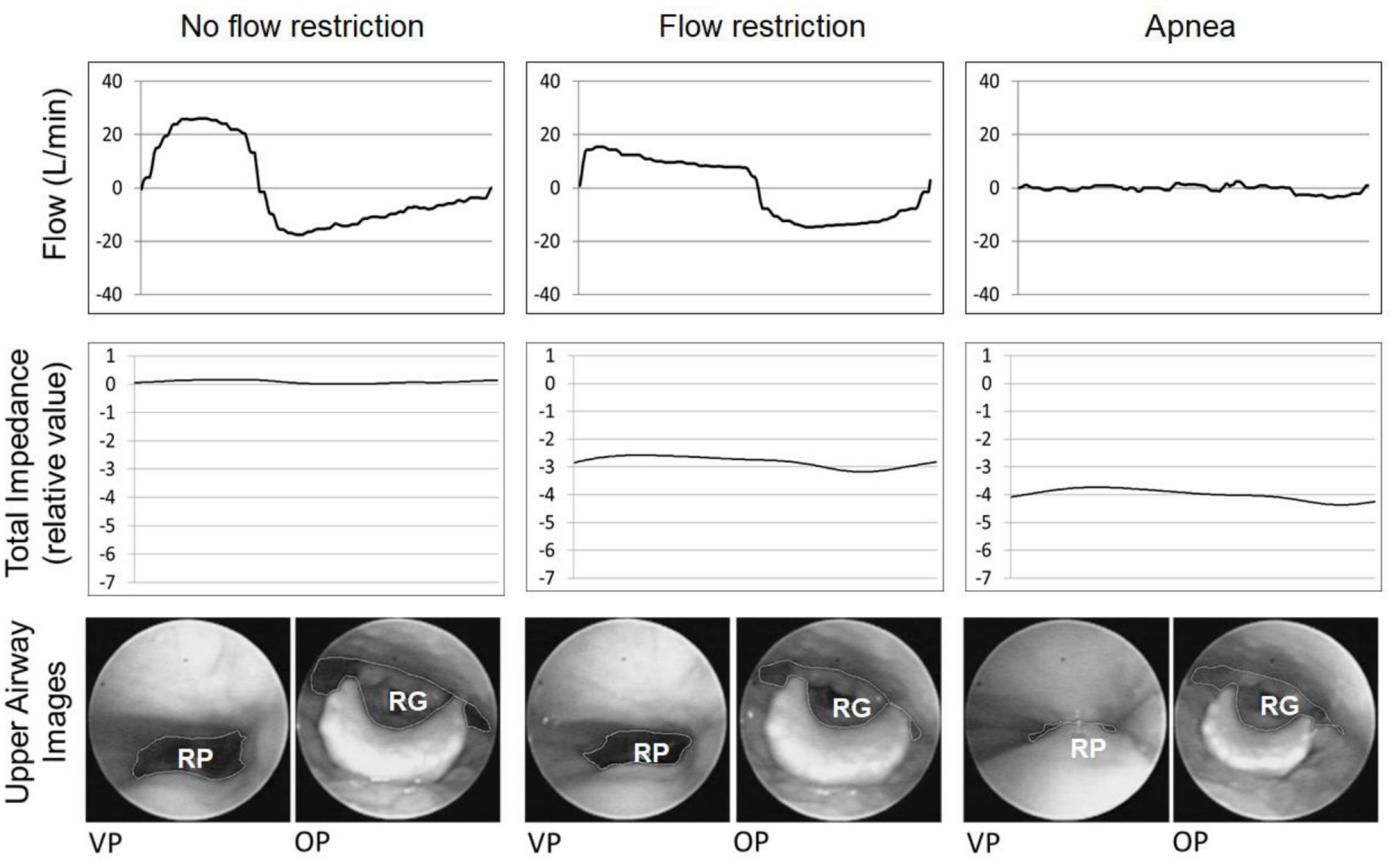
This figure shows an example of a representative respiratory cycle of one subject subjected to different CPAP levels. From left to right: no flow restriction, flow restriction and apnea. From top to bottom: flow curve (dashed line), total neck impedance (solid line), and upper airway images at the velopharynx (VP) and oropharynx (OP) areas. The impedance decreased progressively from no flow restriction to flow restriction and apnea. The chart scale does not allow us to visualize the impedance changes during the breathing cycle. RP, retropalatal; RG, retroglossal.

The minimum mean VP areas during stable breathing and severe flow restriction (six subjects that performed nasofibroscopy) were 20.52 mm^2^ (3.59 SE, range of 12.90–37.18 mm^2^) and 4.03 mm^2^ (2.84 SE, range of 0.52–18.13 mm^2^), respectively (Figure 6). The minimum mean OP areas during stable breathing and during severe flow restriction were 18.50 mm^2^ (3.81 SE, range of 5.53–34.27 mm^2^) and 5.19 mm^2^ (2.36 SE, range of 0.78–15.06 mm^2^), respectively. The GEE analysis indicates that there is a significant VP area effect when controlling for the impedance signal at retroglossal area (*p* = 0.13), with a prevalence ratio of 0.29. Also, the results indicate that there is a significant OP area effect when controlling for the impedance signal at retropalatal area (*p* = 0.11), with a prevalence ratio of 0.45.

**Figure 7:**
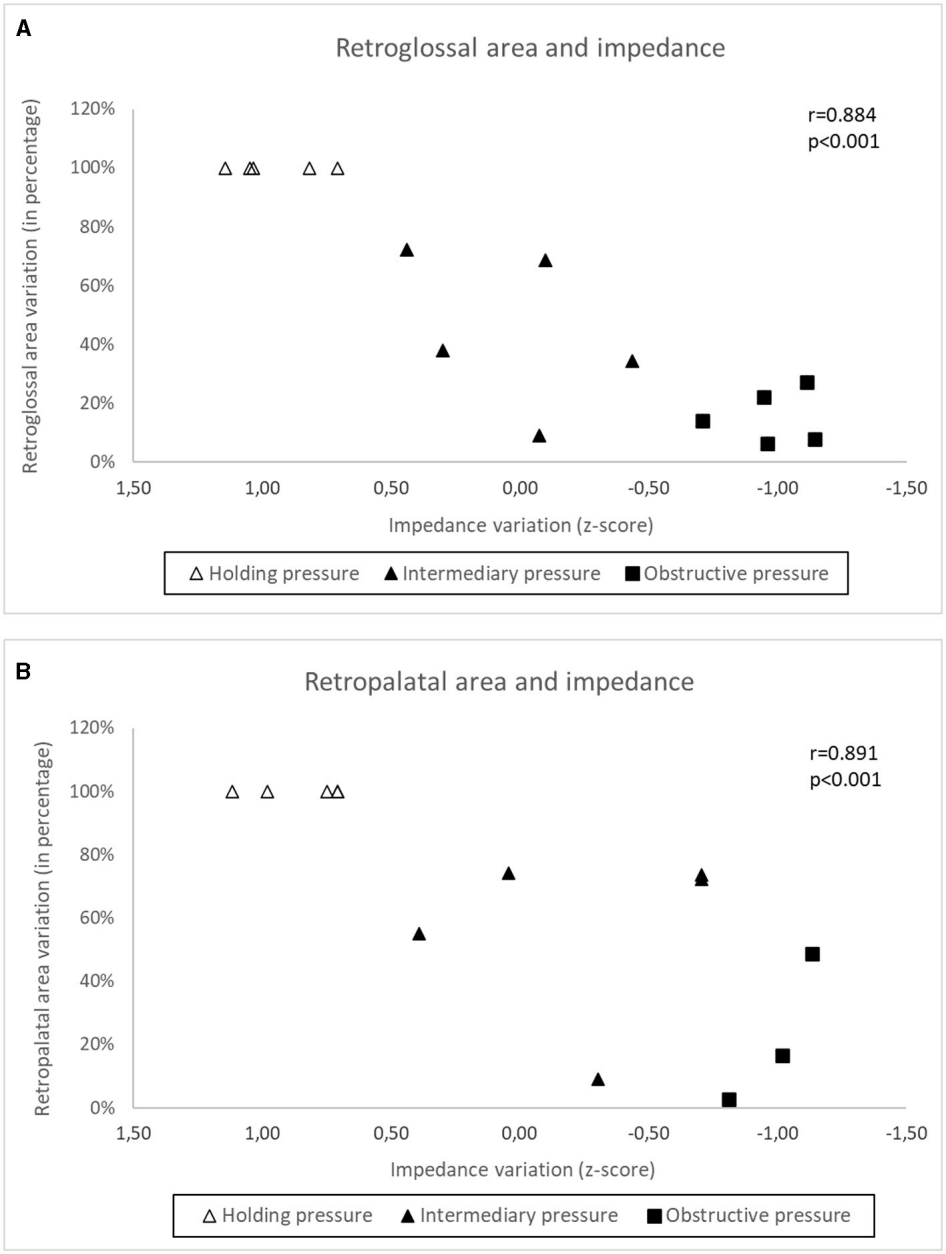
In a subgroup of subjects **(A)** shows the relationship between impedance variation and retropalatal area variation at the holding pressure (no respiratory flow restriction), intermediary pressure (~50% of respiratory flow restriction) and obstructive pressure (apnea). **(B)** Shows the same relationship between impedance variation and retroglossal area variation.

**Figure 8:**
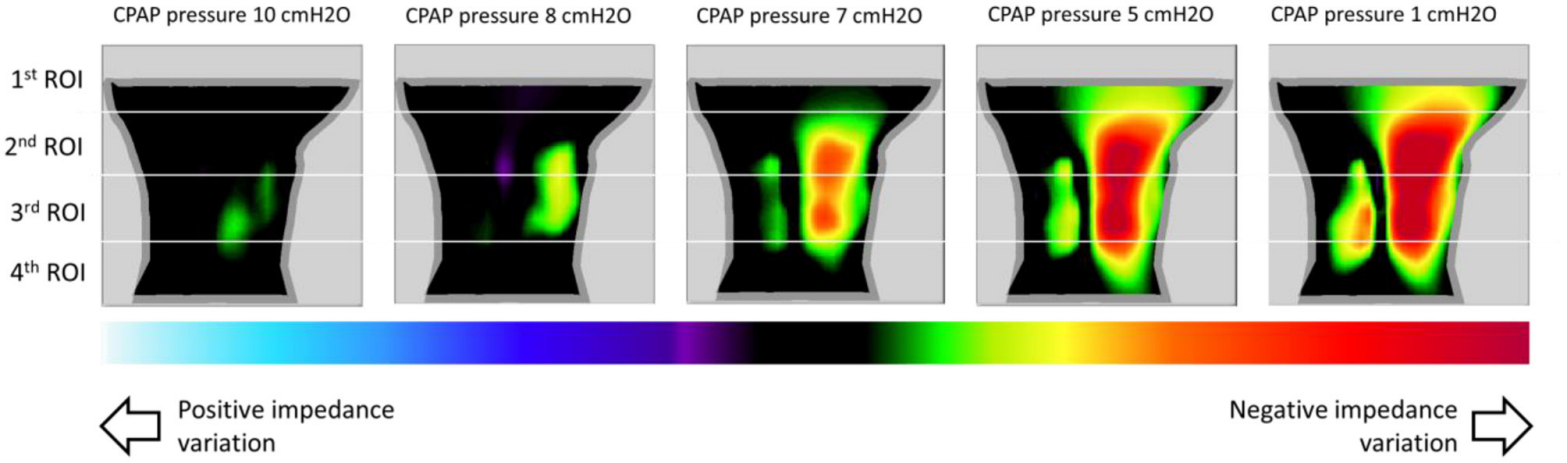
This figure shows images of impedance variation of the sagittal plane, in a representative subject, at different CPAP pressures (pressure of 10 cmH_2_O was used as reference). The images show a decrease in impedance as the CPAP pressure decreases to Pcrit (1 cmH_2_O). There is an artifact near the back of the neck, likely due to differences between the model's shape and the actual shape of the neck. Also, as showed in Table 2, we can observe that negative impedance variation was higher in the 2nd ROI (region of interest).

The group data correlations among the minimum VP and OP areas and total neck impedance were 0.884 and 0.891, respectively (*p* < 0.001) (Figure 7). At Figure 8 it is possible to observe, in a representative subject, that upper airway changes are also visible on the neck electrical impedance tomography image.

## Discussion

This was the first study using EIT method in sleep induced patients. In our study, EIT consistently detected upper airway occlusion induced by applying variable levels of nasal pressure during induced sleep (method of Pcrit determination). There were no differences by gender in our study (regarding baseline characteristics, sleep study, CPAP holding pressure and Pcrit). Also, we didn't notice any sex-specific differences in male vs. female on impedance data acquisition.

Neck EIT was sensitive and varied in concert with the respiratory cycle (Figure 3). Neck EIT also decreased in concert with and in proportion to the level of CPAP reduction. The image of one representative patient (Figure 4) was confirmed via the demonstration of a close relationship between V'Imax and simultaneous neck impedance in all patients (Figure 5). Finally, the upper airway image obtained by direct visualization both on the VP and OP areas correlated with total neck impedance.

We placed the electrodes around the neck in two parallel rings in a zigzag configuration to obtain information from a wide region (that would encompass the pharynx). The zigzag electrode positioning allowed for the construction of a 3D finite element mesh that corresponded to a longitudinal area of 18.4 cm (~9.2 cm rostral and 9.2 cm caudal to the electrode center). Using this configuration neck EIT captured changes in pharyngeal patency cephalic to the electrodes positioned at the neck.

Total neck impedance was sensitive and varied in concert with the tidal volume during stable breathing. We found a strong inverse cross-correlation between the flow and impedance curves (r = −0.817, *p* < 0.001), indicating that impedance valleys (lowest upper airway cross-sectional areas) occurred during the inspiratory phase and that impedance peaks (highest upper airway cross-sectional areas) occurred during the expiratory phase (Figure 3).

These findings are consistent with previous studies of non-invasive continuous imaging of the upper airway during natural sleep, conducted for OSA patients, using the EIT technique (Ayoub et al., 2019, 2020; Kim et al., 2019).

In our study, the high sensitivity of neck EIT, which varied in concert with CPAP reductions, was self-evident in one representative example (Figure 4). Group data derived from all subjects demonstrated a high correlation coefficient between V'Imax and total neck impedance variation (*r* = 0.866). Therefore, the progressive flow restriction associated with progressive CPAP reduction was associated with progressive decreases in neck impedance (indicating a smaller cross-sectional area). This observation is consistent with those of previous studies that demonstrated strong correlations between CPAP levels and pharynx areas (Launois et al., 1993; Morrison et al., 1993; Isono et al., 2002). Additionally, Isono et al. (1997) demonstrated that variable and controlled levels of flow limitation correlate with upper airway cross-sectional area. The assumption that neck EIT correlates with upper airway patency was further confirmed via direct visualization of the upper airway. Also, at Figures 6, 7 we show the same relationship between impedance level, flow restriction and upper airway collapse.

Finally, the finding that the 2nd ROI (rather than the first and most cranial ROI) exhibited the largest variation in impedance during upper airway obstruction (Table 2) clearly indicates that EIT captured changes in pharyngeal patency cephalic to the electrodes positioned on the neck.

### Limitations

Our new method has limitations. A virtual 3D finite element mesh was created based on CT-scan images from a single male volunteer. However, we studied a wide range of cranium, upper airway, soft tissue, and body characteristics (Table 1), and the signals were clear in all subjects. The method may also be applied using customized meshes according to individual biotypes. Additionally, the system only responds to changes in impedance; therefore, we are not able to provide absolute values for anatomical evaluations. Also, Kim et al. (2019) in their study concluded that changes in the upper airway size can be estimated with good accuracy, but shape estimation needs future improvements in the EIT image quality (Kim et al., 2019). We believe that electrical impedance may provide absolute values for anatomical descriptions in the future (Costa et al., 2009b).

Additionally, neck EIT images were obtained with the subjects in a stable position. Ayoub et al. (2019) found that EIT data from the lower face were contaminated by artifacts from respiratory motions, blood flows in the carotid artery and neck movements. In our study, EIT artifacts generated by body motion was limited to controlled conditions but is an important consideration that needs to be resolved in time to come

Although our method was able to assess impedance variations in different upper airway segments, the model of the electrical impedance tomography system in this study was built based on the anatomical characteristics of a single individual. This certainly limited our analysis to the comparison of different anatomical sites and made us prioritize the total impedance analysis. However, we demonstrated that the technique allows segmental evaluation, which we hope will be viable in the future.

## Conclusion

Neck EIT is a sensitive method that varies with the breathing cycle and correlates with peak flow under flow limitation, indicating that neck EIT monitors pharyngeal patency during sleep. Therefore, neck EIT is a promising non-invasive method that may provide insights on the dynamic of upper airway obstruction during sleep.

## Data availability statement

The raw data supporting the conclusions of this article will be made available by the authors, without undue reservation.

## Ethics statement

The studies involving humans were approved by Hospital das Clínicas Ethics Committee (protocol number: 0748/11). The studies were conducted in accordance with the local legislation and institutional requirements. The participants provided their written informed consent to participate in this study.

## Author contributions

VP planned the project, recruited subjects, designed, and performed the study, performed statistical analyses, analyzed the data, and wrote the manuscript. EC planned the project, designed experiments, interpreted the results, and wrote the manuscript. VT, RA, FS, PS, and FM enrolled subjects and performed experiments. MG designed and performed experiments. PG, CC, and MA planned the project, designed experiments, and analyzed the data. GL-F planned the project, designed experiments, analyzed the data, and wrote the manuscript. All authors reviewed, revised, and approved the manuscript for submission.
